# Gas-phase hydrolysis of triplet SO_2_: A possible direct route to atmospheric acid formation

**DOI:** 10.1038/srep30000

**Published:** 2016-07-15

**Authors:** D. James Donaldson, Jay A. Kroll, Veronica Vaida

**Affiliations:** 1 Department of Chemistry, University of Toronto, Toronto, ON Canada; 2 Department of Physical and Environmental Sciences, University of Toronto, ON Canada; 3 Department of Chemistry and Biochemistry, University of Colorado, Boulder, CO USA

## Abstract

Sulfur chemistry is of great interest to the atmospheric chemistry of several planets. In the presence of water, oxidized sulfur can lead to new particle formation, influencing climate in significant ways. Observations of sulfur compounds in planetary atmospheres when compared with model results suggest that there are missing chemical mechanisms. Here we propose a novel mechanism for the formation of sulfurous acid, which may act as a seed for new particle formation. In this proposed mechanism, the lowest triplet state of SO_2_ (^3^B_1_), which may be accessed by near-UV solar excitation of SO_2_ to its excited ^1^B_1_ state followed by rapid intersystem crossing, reacts directly with water to form H_2_SO_3_ in the gas phase. For ground state SO_2_, this reaction is endothermic and has a very high activation barrier; our quantum chemical calculations point to a facile reaction being possible in the triplet state of SO_2_. This hygroscopic H_2_SO_3_ molecule may act as a condensation nucleus for water, giving rise to facile new particle formation (NPF).

Sulfur compounds have been observed in the atmospheres of a number of planets and moons in our solar system including Venus, Earth, Mars, and Io^1^,^2^,^3^ and are known to be important to the evolution of planetary atmospheric chemistry and climate. Photochemical models have been used to explain observations of sulfur in the atmosphere of Earth^4^. Additionally, the models have more recently been applied to ground based observations and measurements from orbiters such as Venus Express^5^,^6^,^7^,^8^,^9^, attempting to explain observations of H_2_SO_4_ and of sulfur oxides (SO, SO_2_, SO_3_) in the middle atmosphere of Venus, a natural laboratory in which to study sulfur chemistry^10^. However, several discrepancies have arisen between the models and observations. For example, high SO_2_ mixing ratios are observed above 90 kilometers in the Venusian atmosphere exceeding model predictions by orders of magnitude^9^. Red light overtone pumping photochemistry of H_2_SO_4_^11^ was previously applied to explain sulfur dioxide profiles and aerosol in Earth’s atmosphere^12^,^13^ and has been included in the models of the atmosphere of Venus^14^. While this chemistry was sufficient to explain SO_2_ profiles on Earth, it does not fully explain the observed sulfur oxide profiles on Venus.

In an oxidizing atmosphere, SO_2_ is transformed to highly hygroscopic sulfuric acid, H_2_SO_4_. Because of its hygroscopicity and ubiquitous nature in Earth’s atmosphere, gas phase sulfuric acid is considered to be a critical agent in much of atmospheric new particle formation (NPF)^15^,^16^. Its gas-phase formation is via the reaction of OH with SO_2_, with the resulting HOSO_2_ species rapidly reacting with atmospherically abundant O_2_, to form SO_3_, whose hydrolysis reaction with water forms H_2_SO_4_. Two water molecules, perhaps as a water dimer, are required for this last process, giving rise to a strong temperature (T)- and relative humidity (RH)-dependent hydrolysis rate^17^,^18^,^19^,^20^. Once formed, the very hygroscopic sulfuric acid molecule provides a good nucleus for the formation of small water clusters, which then may grow by rapid condensation of further water molecules onto this nucleus. The presence of ammonia or small amines in the Earth’s atmosphere is believed to further aid the water condensation process by reducing the water activity in the growing condensation nucleus^15^,^16^,^21^,^22^.

The importance of sunlight to this process is well established: new particle formation is seen to occur primarily during daylight hours^16^. Photochemical NPF in Earth’s lower atmosphere, which has available light at wavelengths > ~280 nm only, cannot involve direct SO_2_ photodissociation because the threshold wavelength for the dissociation SO_2_ → SO + O lies near 217 nm (551 kJ/mol), Rather, as suggested by the mechanism presented above, photons are required for the production of OH radicals that initiate the oxidation SO_2_ to H_2_SO_4_ in the presence of water. Therefore, according to the standard mechanism, for NPF to take place several requirements must be met: At a minimum, there must be sufficient OH to oxidize the SO_2_ and there must be a sufficient concentration of water vapor, at a low enough temperature to produce H_2_O dimers and then hydrolyze SO_3_ to H_2_SO_4_; additionally, the process is aided by a source of ammonia or some other alkaline gas.

In the following we use results of quantum chemical calculations to outline a novel reaction for SO_2_ with water and test this proposed mechanism against a laboratory experiment of particle formation. Theoretical approaches have proven useful in exploring the reactions of SOx-H_2_O systems (see, e.g.)^10^,^14^,^15^. The mechanism involves the near-UV photoexcitation to the lowest forbidden triplet state (^3^B_1_) of SO_2_, either directly, or via intersystem crossing from the allowed excited singlet state (^1^B_1_), which lies at somewhat higher energy. The triplet state molecule then reacts with water, along a barrierless pathway, forming ground state H_2_SO_3_. A singlet-triplet surface crossing in the entrance channel to the reaction ensures a rapid evolution to ground state products. If this mechanism is correct, it suggests the possibility that this chemistry potentially plays a role in NPF in the atmosphere. The H_2_SO_3_ product could conceivably act as a nucleus for water condensation. Experiments in which mixtures of gas phase water and SO_2_ are illuminated with visible-near UV light (at wavelengths too long, λ > 280 nm, for significant OH formation) show rapid NPF when the gas mixture is illuminated, with no NPF observed in the absence of water or light.

## Results

**Figure 1:**
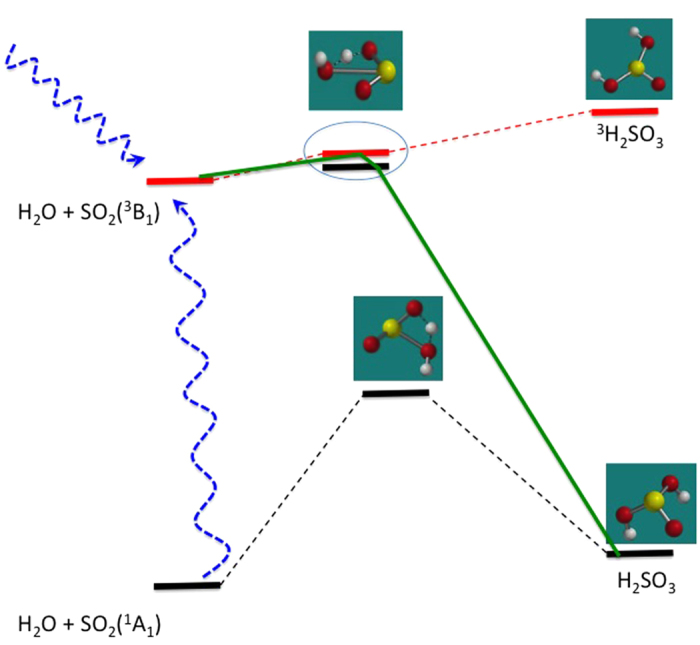
Calculated energetics (at the CCSD/6-311++G(3df,3pd) level) of the SO_2_-H_2_O system. Singlet pathways are shown in black and triplets are in red. The geometries of some key stationary points are shown. The proposed mechanism is illustrated as the green line. It begins on the triplet surface, accessed as indicated by the blue arrows, either by direct excitation or intersystem crossing from a singlet, then switches to the singlet (in the region shown by the circle) then proceeds to the ground state product.

Briefly, we propose that a triplet-state SO_2_ molecule, containing approx. 3 eV more energy than the ground state, may collide with a water molecule in a way which accesses a surface crossing to the sulfurous acid product on the ground state singlet potential energy surface. The structures and energies of the key chemical species, as calculated at the CCSD/ 6-311++G(3df,3pd) level, are illustrated in Fig. 1. Full results of the calculations at this level, as well as those of a preliminary scan at a lower level of theory are given in Tables S1 (for the lower level) and S2 of the Supplementary Information (SI), with reaction energetics reported in Table S3.

In good agreement with previous studies from this group^23^,^24^ and others^25^,^26^, the zero-point corrected energetic barrier to the simple hydrolysis reaction on the ground state (singlet) potential energy surface is calculated to lie 34.8 kcal/mol above reagents. Also in agreement with previous work, the overall reaction is calculated to be endoergic, with a (zero-point corrected) △E_o_° = +5.6 kcal/mol. These values fall well within the range of those previously reported^10^,^11^,^12^,^16^, providing some confidence in the energetics calculated at this level of theory.

We calculate that the lowest-energy triplet state of SO_2_ is of ^3^B_1_ symmetry, with a somewhat larger S-O bond length (1.499 Å vs. 1.443 Å) and a somewhat larger O-S-O angle (125.8° vs. 118.7°) than those calculated for in the ground ^1^A_1_ state. These geometries are in excellent accord with those reported experimentally^27^. At its equilibrium geometry the ^3^B_1_ state is calculated to lie 71.8 kcal/mol above the ground state; the calculated vertical excitation energy lies at 99.4 kcal/mol (corresponding to a maximum in absorption of 288 nm). The triplet state energy is in very good accord with the experimental adiabatic energy difference (73.8 kcal/mol)^27^,^28^. The transition state connecting triplet-state reactants and products lies at low energy – about 6.5 kcal/mol above the reagents – and may be achieved by the approach of a water molecule in such a way that the oxygen and one hydrogen of water lie in the plane of the triplet SO_2_, and bisect the O-S-O angle, which closes slightly, from 126° to 106°, while the O-S bond lengths increase slightly, to 1.54 Å. The distance between the oxygen of water and the sulfur at the calculated transition state is 2.88 Å, compared to those in ground state H_2_SO_3_ of 1.63 Å for the HO-S distances and 1.46 Å for the S = O distance. The fairly low calculated barrier suggests that it might only require a very gentle encounter between a water molecule and a triplet SO_2_ to achieve this TS geometry. Indeed, whether the ^3^B_1_ state is accessed directly, via a vertical excitation, or via intersystem crossing from the (allowed) excited ^1^B_1_ singlet state (at 79.8 kcal/mol excitation)^29^, it will initially contain sufficient energy to overcome the barrier.

At this geometry, the energy of the singlet ground state surface is calculated to lie 5.4 kcal/mol below that of the triplet - a close singlet-triplet encounter. Strong spin-orbit interactions are known to couple singlet and triplet states in SO_2_^28^,^29^; since water is known to be an efficient quencher of the SO_2_ triplet state^30^ it follows that collisions with water molecules may induce efficient intersystem crossing. Thus it seems likely that the triplet SO_2_-H_2_O system could access the singlet surface, which could then evolve towards H_2_SO_3_ product. From the triplet transition state a simple rotation of the SO_2_ moiety “away” from the water will transform the geometry to that of ground state H_2_SO_3_. This pathway is illustrated in Fig. 1.

If the system evolves to singlet products as we postulate, the resulting sulfurous acid is calculated to exhibit strong attraction towards water, with zero-point corrected binding energies of 7.7, 7.9 and 6.2 kcal/mol for the addition of one, two and three water molecules, respectively at a lower level of calculation, B3LYP/6-311+G(2df,2p) (see Table S4 for details). The binding energy to a single water molecule is about three times that calculated for the water dimer at the same level of theory (2.65 kcal/mol), also given in the SI. If it is formed, H_2_SO_3_ is therefore likely to act as a condensation nucleus for water, and could then participate in NPF.

**Figure 2:**
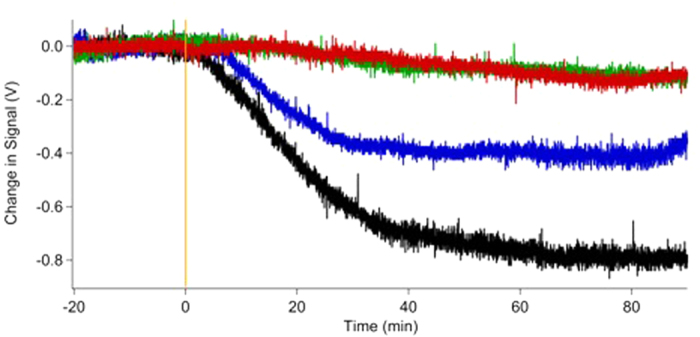
Results of a scattering experiment in which either pure samples or mixtures of SO_2_ and water were illuminated starting at time = 0. The scattered light intensity is displayed as a voltage vs. time during the experiment. The red and green traces show the scattering observed from 7.010 Torr of water and 3.015 Torr of SO_2_, respectively. The blue trace shown is for a mixture of 7.010 Torr of water and 0.891 Torr of SO_2_, and the black trace shows scattering from a mixture of 7.017 Torr water and 3.3 Torr SO_2_. No scattering is observed in the absence of light (−20–0 min); only when SO_2_ and H_2_O are both present is scattering (indicating particle formation) observed upon illumination (0–90 min). The amount of scattering is greater with higher initial SO_2_ partial pressure.

We test this chemistry by performing an experiment in which a mixture of either 3 or 1 Torr of SO_2_ with 7 Torr of H_2_O is introduced into a glass flow cell, then illuminated with a Xe arc lamp through a 295 nm long-pass filter. (See Figure S1 in the SI for absorption spectra of the mixture and the filter transmission). Light scattering from aerosols is observed immediately upon illumination of the SO_2_ + H_2_O mixture and is recorded by measuring the decrease in transmitted intensity of a 532 nm laser beam passed through the cell. In Fig. 2 we display a typical result from such experiments: Illumination of either SO_2_ or H_2_O alone by the arc lamp does not induce light scattering, but illumination of the gas mixture causes a rapid appearance of particles in the flow cell, as indicated by the onset of strong light scattering. No scattering of the probe laser is observed in the absence of illumination.

It is highly unlikely that NPF is induced in this experiment via OH-mediated oxidation of SO_2_ in the presence of water. There is no oxygen (or ozone) present in the reaction cell and the 295 nm long-pass optical filter (1% transmission at 276.5 nm) removes any UV radiation with sufficient energy to initiate bond-cleavage in SO_2_ or H_2_O; therefore radical formation is highly unlikely. We tested this expectation by introducing cyclohexane, an OH scavenger, into the cell; this is expected to reduce or eliminate any OH-mediated reactions taking place there. In the absence of SO_2_, the addition of C_6_H_12_ did not show any effect, either in the light or the dark, with or without water present, demonstrating the lack of OH chemistry in these experiments. However, when SO_2_ was also present, particle formation was seen under illumination, even without water (see Figure S2). We suggest that this observation arises from the highly reactive nature of ^3^SO_2_ with organics^31^, which would give rise to radical condensation type reactions in the cell. Any heating due to light absorption in the cell would be expected to mitigate NPF, so small temperature increases are also unlikely to be responsible for the observed particle formation. However, the light transmitted by the filter does overlap with the near-UV absorption spectrum of SO_2_ (as displayed by Figure S1 in SI), making SO_2_^*^-mediated photochemical processes possible.

When SO_2_ is excited into the manifold of states accessible in the near-UV spectral region it is known to undergo rapid and irreversible decay into lower-lying triplet levels^29^. Photochemistry involving S-O bond cleavage is energetically impossible for a single SO_2_ molecule at these excitation wavelengths, so any chemical fate must involve a reaction of excited SO_2_. Although SO_3_ formation via the reaction of triplet SO_2_ with ground state SO_2_ has been reported^32^, the rate constant for this reaction is 20 times smaller than the quenching rate of triplet SO_2_ by water^30^. If NPF in our experiment is due to SO_3_ hydrolysis one expects a quadratic dependence on the SO_2_ concentration. Inspection of the results presented in Fig. 2 shows that this is not the case: increasing partial pressure of SO_2_ by a factor of ~3 results in only a 2-fold increase in the scattering intensity, rather than the order of magnitude change expected for a quadratic dependence.

The rate of collisional quenching of phosphorescence from triplet SO_2_ by water has been reported only once to our knowledge^30^. That report gives a collisional quenching rate constant of 1.4 × 10^−12 ^cm^3^ molec^−1^ s^−1^ - about 1% of the gas-kinetic collisional rate constant, and at least 10 times greater than quenching by N_2_, O_2_ or rare gases^30^. The products of this quenching reaction were not identified, but our observation of NPF clearly supports a chemical process such as the triplet reaction we propose here. A simple kinetic box model of the illuminated SO_2_ - H_2_O system, with relevant rate constants^4^,^20^,^33^, and assuming the quenching of ^3^SO_2_ by water^30^ to be totally due to reaction, shows that H_2_SO_3_ formation in our cell (with p_H_2_O_ = 7 Torr) dominates over H_2_SO_4_ when the SO_2_ partial pressure is less than about 2.4 Torr. These results are presented in the SI.

## Discussion

In the above, we have demonstrated a novel photochemical mechanism for the formation of sulfurous acid, an unstable molecule that has not been isolated in the gas phase. One consequence of this chemistry is the formation of new particles from mixtures of illuminated SO_2_ and water in the absence of gas phase oxidants. We suggest that the previously reported, efficient quenching of triplet state SO_2_ by water^30^ may proceed to some extent via chemical reaction to form hygroscopic sulfurous acid directly. However, this species is highly unstable^23^,^24^,^25^,^34^, and has not been observed conclusively, either in the gas phase or in solution^34^. Indeed, a modeling study has demonstrated that hydrolysis of SO_3_ is more significant than that of SO_2_ in the atmosphere, in spite of the latter’s much greater abundance^35^. One important reason for this is that, unlike H_2_SO_4_ generated by hydrolysis of SO_3_, hydrolysis of ground state SO_2_ to give H_2_SO_3_ is an endothermic reaction^23^,^24^,^25^,^34^,^35^,^36^. Additionally, as with the hydration of SO_3_, there is a high barrier to H_2_SO_3_ formation from SO_2_+H_2_O; this barrier drops somewhat with the participation of more water molecules^23^,^25^,^34^,^36^, but is still too high to overcome at normal atmospheric temperatures. One way to overcome the instability in the gas phase has been proposed recently: the participation of ammonia lowers the barrier for the hydrolysis reaction^25^. However, given that there are at least three molecules required for this process to become energetically viable, the entropic cost of the reaction will increase considerably.

Thus the novel photochemically driven hydrolysis of SO_2_ mechanism reported here is of interest as a potential synthetic route to the elusive sulfurous acid molecule and may also be important in some planetary atmospheres, such as on Venus. To explore when this might be operational in Earth’s atmosphere, we take the upper limit for the ^3^SO_2_ formation rate to be the rate of absorption of actinic photons by ground state SO_2_ (that is, assume that every excited molecule becomes a triplet), and the loss rate of the triplet to be that of triplet deactivation by air. This yields a steady-state fraction of SO_2_ in the triplet state of about 10^−12^ - 10^−11^. Given a concentration ratio of water vapor to OH radicals on the order of 10^10^, the collision rate of OH with SO_2_ is about 10–100 times that of collisions of ^3^SO_2_ with water. Therefore we would expect that SO_3_ formation would dominate under these atmospheric conditions. However, since the hydrolysis of SO_3_ depends on the square of the water vapor concentration^8^,^19^, under conditions of very low humidity, the triplet mechanism may be a substantial contributor to NPF and play some role in Earth’s atmosphere. Indeed, Loerting *et al*.^35^ also suggest that the energetically unfavored hydrolysis of ground state SO_2_ may become as important as SO_3_ hydrolysis under such conditions.

The box model analysis of our experimental results bears this out. Taking an upper limit for the reaction of ^3^SO_2_ with water to be the quenching rate, the box model results in Figure S3 show that sulfurous acid formation dominates over sulfuric acid formation in our experimental setup. Even assuming the reactive quenching to be only 10% of the total only reduces the amount of H_2_SO_3_ to be about half of that of the H_2_SO_4_ produced in the model; that is, the triplet hydrolysis mechanism is still significant. Of course, in Earth’s atmosphere the SO_2_*+SO_2_ reaction will not be important, due to the much lower concentrations of sulfur dioxide. We conclude that, although under some circumstances SO_3_ formation (and thus NPF initiated by sulfuric acid) is certainly possible via reaction of ground-state with excited-state SO_2_ molecules, H_2_SO_3_ is likely a significant contributor to the NPF we observed here, and may play an important role in NPF in other planetary atmospheres where water may be limited such as in the Earth’s upper stratosphere or the middle atmosphere of Venus. In these cases H_2_SO_3_ formation may be favored over the water-catalyzed formation of H_2_SO_4_.

## Methods

Density-functional theory was used to perform initial energy scans of the singlet and triplet reactions, using the Spartan 14 series of programs^37^,^38^ running on a Mac under the OS-X operating system. For this, the geometries were optimized and the energies were calculated at the B3LYP 6-311 + G(2df, 2p) level. Following the identification of the important stationary points, further geometry optimizations were carried out at the B3LYP/6-311++G(3df,3pd) level, with zero-point energies calculated from the predicted harmonic vibrational frequencies without application of a correction factor. Ab-initio single-point energies including configuration interaction were then calculated at these reoptimized geometries using CCSD and the 6-311++G(3df, 3pd) basis set. The overall accuracy of the energetics is assumed to be similar to that reported in Fu *et al*.^39^.

The experimental setup consisted of a glass cell with quartz windows evacuated using an Edwards 12 E2-M12 dual stage rotary vane mechanical pump. The cell was a 90 degree angle square cross with a 2.5 inch diameter and 8 inch long pathlength. It was pumped out overnight to remove impurities or potential contaminants and then filled with the sample of interest (SO_2_, H_2_O or a mixture of the two) to p = 7.010 Torr water, degassed by 3–5 freeze pump thaw cycles. A sulfur dioxide (>99.9% purity, Sigma Aldrich) pressure of p = 3.015 Torr was used alone, or a mixture of the two (water p = 7.010 Torr with 3.3 Torr of SO_2_ or water p = 7.017 Torr with 0.891 Torr of SO_2_) was introduced to the cell. The cell was allowed to equilibrate for at least 20 minutes and then was exposed to light from a 450 watt Xe arc lamp (Newport) filtered using a N-WG-295 50 mm diameter, 3 mm thick, longpass optical filter (Edmond Optics) with a stopband limit (0.001% transmittance) of 250 nm, passband limit of 400 nm, and a cut-off position (50% transmittance) of 300 ± 6 nm. Scattering data was collected using a Brightline Pro green dot projecting alignment laser (Laserglow Technologies) detected by a PDA155 amplified silicon photodetector (Thorlabs). The laser was directed through an iris and along the 8 inch pathlength of the cell, through a second iris, and then detected using the photodetector.

## Additional Information

**How to cite this article**: Donaldson, D. J. *et al*. Gas-phase hydrolysis of triplet SO_2_: A possible direct route to atmospheric acid formation. *Sci. Rep.* **6**, 30000; doi: 10.1038/srep30000 (2016).

## Supplementary Material

Supplementary Information
