# Supplementary material for: Gas-phase hydrolysis of triplet SO2: A possible direct route to atmospheric acid formation
Source: Sci Rep. 2016 Jul 15;6:30000. doi: 10.1038/srep30000 (PMC4945918; doi:10.1038/srep30000)

Supplementary Information for "Gas-phase hydrolysis of triplet SO_2: A possible direct route to atmospheric acid formation"

D. James Donaldson,[1,2*] Jay A. Kroll[3] and Veronica Vaida[3]
1) *Department of Chemistry, University of Toronto, Toronto, ON Canada*
2) *Department of Physical and Environmental Sciences, University of Toronto, ON Canada*
3) *Department of Chemistry and Biochemistry, University of Colorado, Boulder, CO USA*

Table S1: Results of quantum chemical calculations using 6-311+G(2df,2p) basis set

**species**	**E @ B3LYP (hartree)**	**E @ CCSD (hartree)**	**ZPE (kcal/mol)**
SO_2 (ground state)	-548.707561	-547.928766	4.34
$^3SO_2$	-548.603258	-547.813177	2.87
H_2O	-76.4625619	-76.3205046	13.39
^1TS	-625.121220	-624.1906869	17.63
^3TS	-625.063342	-624.1232155	16.17
singlet at ^3TS geometry	-625.065454	-624.1293992	16.36
ground state H_2SO_3	-625.166102	-624.2456777	20.15
$^3H_2SO_3$	-625.052089		17.36
H_2SO_3-H_2O complex	-701.644812		35.96
H_2SO_3-$(H_2O)_2$ complex	-778.124250		52.06
H_2SO_3-$(H_2O)_3$ complex	-854.600499		67.80
$(H_2O)_2$ complex	-152.933044		29.11

Table S2: Results of quantum chemical calculations using 6-311++G(3df,3pd) basis set

**species**	**E @ B3LYP (hartree)**	**E @ CCSD (hartree)**	**ZPE (kcal/mol)**
SO_2 (ground state)	-548.715803	-547.944418	4.31
$^3SO_2$	-548.609761	-547.828188	3.24
H_2O	-76.4645109	-76.3291151	13.37
^1TS	-625.132133	-624.218208	17.79
^3TS	-625.070660	-624.146233	16.15
singlet at ^3TS geometry	-625.074792	-624.155358	16.35
ground state H_2SO_3	-625.175065	-624.268640	20.23

Table S3: Zero-point corrected reaction energetics at the B3LYP (CCSD) levels using the 6-311++G(3df,3pd) basis set.

Reaction	ΔE *zero-point corrected* (kcal/mol)
$^1SO_2 + H_2O$ --> ^1TS	+30.3 (+34.8)
$^1SO_2 + H_2O$ --> $^1H_2SO_3$	+5.8 (+5.6)
$^1SO_2$ --> $^3SO_2$	+65.4 (+71.8)
$^3SO_2 + H_2O$ --> ^3TS	+1.8 (+6.48)
ΔE (^1TS - ^3TS)	-2.2 (-5.4)

Table S4: Reaction energetics at the B3LYP (CCSD) levels using 6-311+G(2df,2p) basis set

Reaction	ΔE (kcal/mol)	ΔE *zero-point corrected* (kcal/mol)
$^{1}SO_2 + H_2O$ --> ^{1}TS	+30.7 (+36.8)	+30.6 (+36.6)
$^{3}SO_2 + H_2O$ --> ^{3}TS	+1.55 (+6.57)	+1.45 (+6.48)
$^{1}SO_2 + H_2O$ --> $^{1}H_2SO_3$	+2.5 (+2.25)	+5.0 (+4.64)
$H_2SO_3 + H_2O$ --> H_2SO_3 -(H_2O)	-10.1	-7.7
H_2SO_3 -(H_2O)+ H_2O --> H_2SO_3 -$(H_2O)_2$	-10.6	-7.9
H_2SO_3 -$(H_2O)_2$ + H_2O --> H_2SO_3 -$(H_2O)_3$	-8.6	-6.25
$H_2O + H_2O$ --> $(H_2O)_2$	-4.97	-2.65

Figure S1: Near-UV spectra of $SO_2(g)$ and long-pass optical filter used in the experiments

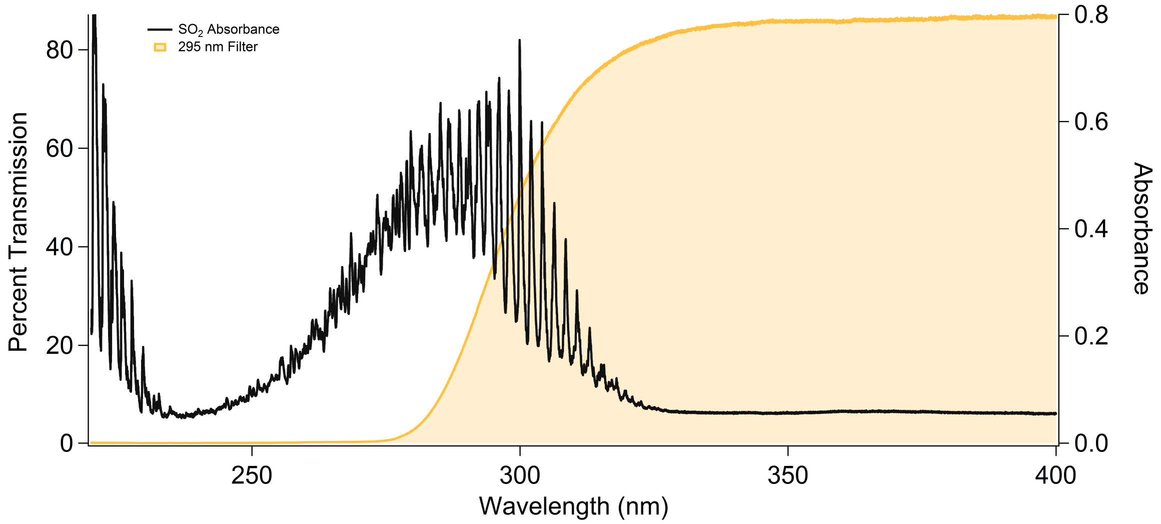

Figure S2: Particle formation following illumination of gas phase SO_2 + cyclohexane mixture in the absence of water.

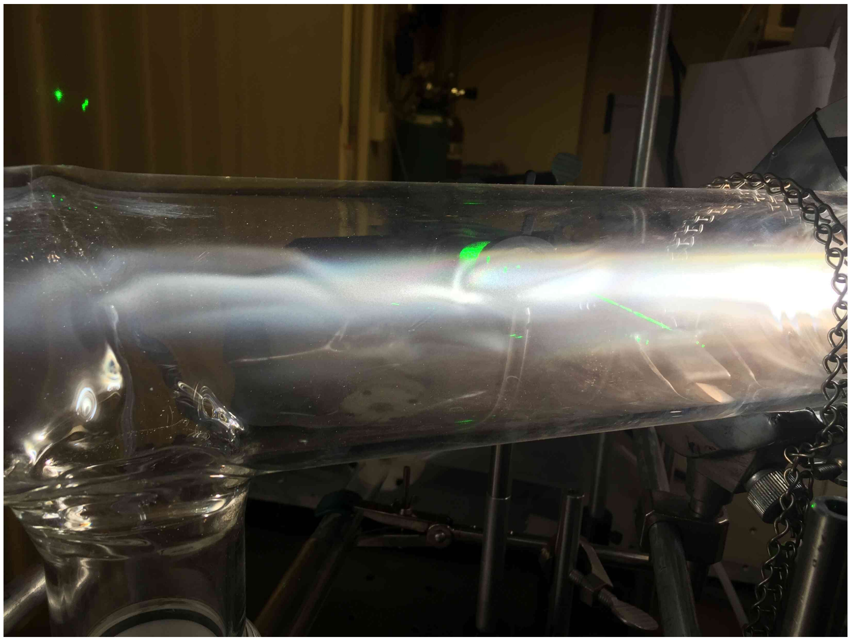

Chemical box model description

A chemical box model employing the mid-point method for integration was used to model the photochemistry of SO_2 in our cell. The reactions included and their rate constants are found in the table below. The rate constant for the photo-excitation of SO_2 was approximated for our setup using a 450 W xenon arc lamp as the source for photo-excitation. The rate constant for the formation of sulfurous acid is taken to be the value reported by Sidebottom et al. for deactivation of $^3SO_2$ through collisions with water molecules. Additionally, steady state approximations for the concentrations of $^1SO_2$, $^3SO_2$, and SO_3 were employed in the box model. All calculations using the box model employed a time step of 0.1 seconds and were carried out to a time of 180 minutes. Using this simple box model to understand the reactions in our experimental setup we see that sulfurous acid formation dominates and is the major component of sulfur acid formation predicted. Under conditions with a greater partial pressure of SO_2 there is an increase in collisions between ground state and electronically excided SO_2 leading to a greater formation of SO_3 and thus sulfuric acid. Even so, our box model predicts that under our experimental conditions the majority of acid formed is sulfurous acid.

Table S3: Reactions and rate constants used in box model of the experimental setup.

Reaction	Rate Constant	Source
SO_2 + hv → $^1SO_2$	k_1= 3.2E-5	Approximation for our setup
$^1SO_2$ → SO_2 + hv	k_2 =2.2E4	reference 1
$^1SO_2$ → $^3SO_2$	k_3=1.5E3	reference 1
$^1SO_2$ + M → SO_2 + M	k_{4a} =1.0E-11 (M=SO_2)	reference 1
$^1SO_2$ + M → SO_2 + M	k_{4b} = 2.9E-11 (M≠SO_2)	reference 1
$^1SO_2$ + M → $^3SO_2$ + M	k_{5a} =1.0E-12 (M=SO_2)	reference 1
$^1SO_2$ + M → $^3SO_2$ + M	k_{5b} = 3.0E-11 (M≠SO_2)	reference 1
$^1SO_2$ + SO_2 → SO_3 + SO	k_6 = 4.0E-12	reference 1
$^3SO_2$ → SO_2 +hv	k_7 = 1.1E3	reference 1
$^3SO_2$ + M → SO_2 + M	k_8 = 5.8E-13 (M=SO_2)	reference 1
$^3SO_2$ + SO_2 → SO_3 + SO	k_9 = 7E-14	reference 1
SO_3 +H_2O → H_2SO_4	k_{10} = 2.31907576 E-31 * $[H_2O]^2$	reference 2 (T=298K) **Pseudo first order with respect to [SO_3]
$^3SO_2$ + H_2O → H_2SO_3	k_{11}=1.4E-12	Adapted from reference 3

References

1. Whitehill, A. R.; Ono, S., Excitation band dependence of sulfur isotope mass-independent fractionation during photochemistry of sulfur dioxide using broadband light sources. *Geochim. Cosmochim. Acta* **2012,** *94*, 238-253.
2. Lovejoy, E. R.; Hanson, D. R.; Huey, L. G., Kinetics and products of the gas-phase reaction of SO_3 with water. *J. Phys. Chem.* **1996,** *100* (51), 19911-19916.
3. Sidebottom, H. W.; Badcock, C. C.; Jackson, G. E.; Calvert, J. G.; Reinhardt, G. W.; Damon, E. K., Photooxidation of sulfur dioxide. *Environmental Science & Technology* **1972,** *6* (1), 72-79.

Figure S3: Results of box model for (a) p(SO_2)=3.015 Torr and (b) p(SO_2)=0.891 Torr. The red traces indicate the concentration of sulfuric acid (H_2SO_4) formed and the blue traces indicate the concentration of sulfurous acid (H_2SO_3) formed as a function of time following the start of illumination.

(a)

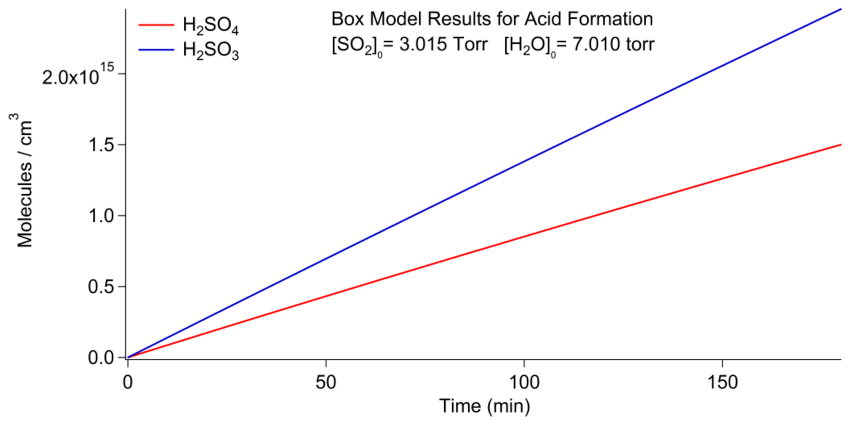

(b)

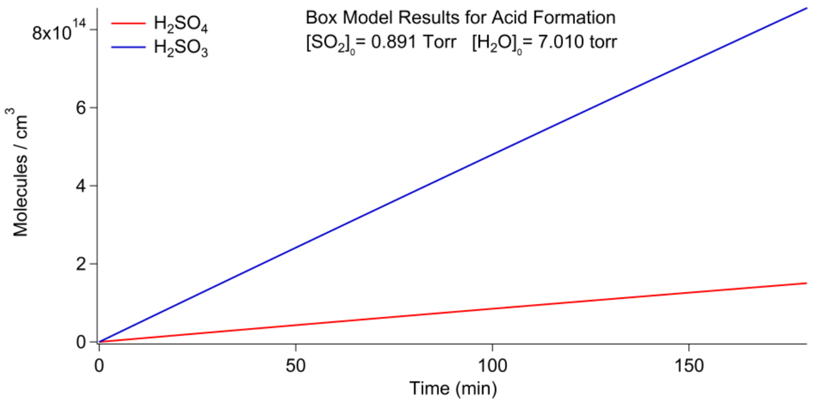

Supplement: Supplementary Information [file srep30000-s1.pdf]
